# Diagnostic Accuracy of Prion Disease Biomarkers in Iatrogenic Creutzfeldt-Jakob Disease

**DOI:** 10.3390/biom10020290

**Published:** 2020-02-12

**Authors:** Franc Llorens, Anna Villar-Piqué, Peter Hermann, Matthias Schmitz, Olga Calero, Christiane Stehmann, Shannon Sarros, Fabio Moda, Isidre Ferrer, Anna Poleggi, Maurizio Pocchiari, Marcella Catania, Sigrid Klotz, Carl O’Regan, Francesca Brett, Josephine Heffernan, Anna Ladogana, Steven J. Collins, Miguel Calero, Gabor G. Kovacs, Inga Zerr

**Affiliations:** 1 Department of Neurology, National Reference Center for CJD Surveillance, University Medical Centre Göttingen, 37075 Göttingen, Germany; 2 Network Center for Biomedical Research in Neurodegenerative Diseases (CIBERNED), L’Hospitalet de Llobregat, 08908 Llobregat, Spain; 3 Bellvitge Biomedical Research Institute (IDIBELL), L’Hospitalet de Llobregat, 08908 Llobregat, Spain; 4 German Center for Neurodegenerative Diseases (DZNE), 37075 Göttingen, Germany; 5 Chronic Disease Programme (UFIEC)-CROSADIS, Instituto de Salud Carlos III, 28029 Madrid, Spain; 6 Network Center for Biomedical Research in Neurodegenerative Diseases (CIBERNED), 28031 Madrid, Spain; 7 Australian National Creutzfeldt-Jakob Disease Registry, Florey Institute, The University of Melbourne, Melbourne 3010, Australia; 8 Fondazione IRCCS Istituto Neurologico Carlo Besta, Unit of Neurology 5 and Neuropathology, 20133 Milan, Italy; 9 Department of Pathology and Experimental Therapeutics, University of Barcelona, L’Hospitalet de Llobregat, 08907 Llobregat, Spain; 10 Department of Neuroscience, Istituto Superiore di Sanità, 00161 Rome, Italy; 11 Institute of Neurology, Medical University of Vienna, Vienna 1097, Austria; 12 Department of Neuropathology, Beaumont Hospital, Dublin 9, Ireland; 13 Department of Medicine (RMH), The University of Melbourne, Melbourne 3050, Australia; 14 Department of Laboratory Medicine and Pathobiology and Tanz Centre for Research in Neurodegenerative Disease, University of Toronto, Toronto, ON M5T 0S8, Canada; 15 Laboratory Medicine Program, University Health Network, Toronto, ON M5G 2C4, Canada

**Keywords:** Iatrogenic Creutzfeldt-Jakob disease, dura matter graft, corneal transplant, growth hormone, biomarker, cerebrospinal fluid, electroencephalogram, magnetic resonance imaging, RT-QuIC

## Abstract

Human prion diseases are classified into sporadic, genetic, and acquired forms. Within this last group, iatrogenic Creutzfeldt–Jakob disease (iCJD) is caused by human-to-human transmission through surgical and medical procedures. After reaching an incidence peak in the 1990s, it is believed that the iCJD historical period is probably coming to an end, thanks to lessons learnt from past infection sources that promoted new prion prevention and decontamination protocols. At this point, we sought to characterise the biomarker profile of iCJD and compare it to that of sporadic CJD (sCJD) for determining the value of available diagnostic tools in promptly recognising iCJD cases. To that end, we collected 23 iCJD samples from seven national CJD surveillance centres and analysed the electroencephalogram and neuroimaging data together with a panel of seven CSF biomarkers: 14-3-3, total tau, phosphorylated/total tau ratio, alpha-synuclein, neurofilament light, YKL-40, and real-time quaking induced conversion of prion protein. Using the cut-off values established for sCJD, we found the sensitivities of these biomarkers for iCJD to be similar to those described for sCJD. Given the limited relevant information on this issue to date, the present study validates the use of current sCJD biomarkers for the diagnosis of future iCJD cases.

## 1. Introduction

Prion diseases or transmissible spongiform encephalopathies (TSEs) are a family of rare neurodegenerative disorders that affect both humans and animals, caused by the conversion of the physiological cellular prion protein (PrP^c^) into a disease-associated isoform (PrP^Sc^). Principal prion disease neuropathological hallmarks are the presence of spongiform changes in the neuropil sometimes associated with massive neuronal loss, neuro-inflammation in the form of astrocytic gliosis and the deposition of aggregated prion protein in the brain parenchyma [1]. In humans, prion diseases can be classified according to their etiology as sporadic, genetic, and acquired [2]. The sporadic form of the disease, also known as sporadic Creutzfeldt-Jakob disease (sCJD), accounts for about 85–90% of all human cases [3]. Some 10–15% of cases are associated with autosomal dominant pathogenic sequence variations in the prion protein gene (*PRNP*), while acquired forms are rare and are caused by the transmission of infective material from human to human (Kuru and iatrogenic Creutzfeldt-Jakob disease (iCJD)) or from cattle to human (variant CJD) [4]. Iatrogenic transmission of CJD occurs through particular surgical and medical procedures. The use of contaminated growth hormone and dura mater grafts derived from human cadavers with undiagnosed sCJD are the principal sources of iCJD [5,6]. Other sources are corneal transplant, treatment with cadaveric pituitary-derived gonadotropin, and the use of CJD-contaminated electroencephalogram (EEG) depth electrodes and neurosurgical instruments. Packed red blood cells from variant CJD donors are another source of secondary infection. Iatrogenic transmission of the CJD agent has been reported in over 490 patients worldwide [7].

iCJD reached its highest incidence in the decade of the 1990s and since then the number of reported cases has drastically decreased [6]. It is not expected that new iCJD cases will arise in the coming years except for cases with very long incubation periods. However, the risk of human-to-human prion disease transmission is still present due to potential new sources of iatrogenic contamination through humans exposed to zoonotic agents or secondary transmissions [8,9,10]. Despite the fact that iCJD diagnosis is based on the presence of a recognized iatrogenic source, unrecognized mechanisms of human prion disease transmission may occur.

Clinical and neuropathological presentation of iCJD is related to the route of exposure to human prions, the transmitted CJD strain, and the *PRNP* genotype of the patient [5,6,11]. Thus, potential singularities in prion disease biomarkers in different iCJD types might be expected. In this regard, there is limited information about the accuracy of prion disease biomarkers, including EEG, neuroimaging, and CSF tests in iCJD. To date, available data in the literature include a limited number of iCJD cases and/or a reduced number of biomarkers employed [12,13,14,15,16].

In the present study, we characterised the biomarker profiles of iCJD, using a cohort generated from seven national prion disease surveillance centres and determined their clinical value in reference to established cut-off points for sCJD.

## 2. Materials and Methods

### 2.1. Patients and CSF Sampling

The study included 23 iCJD cases collected from the following CJD reference centers: 1) Clinical Dementia Center and the National Reference Center for CJD Surveillance at the University Medical Center, Göttingen, Germany (*n* = 11), 2) National Centre of Microbiology-Carlos III Institute of Health, Madrid, Spain (*n =* 2), 3) Istituto Superiore di Sanità, Rome, Italy (*n =* 1), 4) Australian National CJD Registry, The Florey Department of Neuroscience and Mental Health, Melbourne, Australia (*n =* 2), 5) Medical University of Vienna, Austria (*n =* 4), 6) Fondazione IRCCS Istituto Neurologico Carlo Besta, Milan, Italy (*n =* 1), and 7) National CJD surveillance center, Beaumont Hospital, Dublin, Ireland (*n =* 2). Iatrogenic CJD was diagnosed according to established World Health Organization (WHO) criteria [17]. Twenty iatrogenic cases were associated with dura matter grafts, two with human growth hormone, and one with corneal transplantation. Clinical symptoms of the 23 iCJD patients are indicated in Supplementary Table S1. CSF was collected for diagnostic purposes during regular prospective surveillance activities of the participating centers and stored in polypropylene tubes at −80 ºC at each diagnostic centre. For this study, CSF was shipped with dry ice to the University Medical Center of Göttingen to perform additional CSF biomarker tests.

### 2.2. CSF Analyses

The presence of 14-3-3 protein in the CSF was determined with western-blot (WB) according to established CJD diagnostic protocols [18]. Cases with inconclusive outcome (traces of 14-3-3 in the WB) were considered negative. 14-3-3 gamma was quantified using the enzyme-linked immunosorbent assay (ELISA) 14-3-3 gamma from Circulex (Nagano, Japan). Total-tau (t-tau) and phosphorylated tau T181 (p-tau) were quantified using the INNOTEST^®^hTAU-Ag and INNOTEST^®^ PHOSPHO-TAU(181P) ELISA kits from Fujirebio (Ghent, Belgium), respectively. Neurofilament light (Nfl) was quantified using the Uman Diagnostics NF-light assay from Uman Diagnostics (Umeå, Sweden). Alpha-synuclein (a-syn) was quantified using the a-syn ELISA kit from EUROIMMUN as described before [19]. YKL-40 was quantified using the MicroVue YKL-40 ELISA assay from Quidel (San Diego, CA, USA). Real-time quaking-induced conversion (RT-QuIC) was performed as described before [20]. The analysts were blinded to clinical data. CSF biomarkers were centrally quantified (Clinical Dementia Center—Göttingen) at the time of the present study with the exception of WB 14-3-3, which was locally analysed in each of the participants’ laboratories at the time of diagnosis.

### 2.3. Electroencephalogram and Magnetic Resonance Imaging

Electroencephalogram (EEG) and magnetic resonance image (MRI) tests were performed as routine clinical diagnostic studies at each prion diagnostic centre in the framework of epidemiological studies [21,22]. For the present study, biomarker outcomes were reported as “positive” or “negative” according to WHO criteria [17], which include high signal abnormalities in the caudate nucleus and/or putamen on diffusion-weighted imaging (DWI), or fluid attenuated inversion recovery (FLAIR) and the presence of generalized periodic complexes in the EEG.

### 2.4. Genetic Test:

Determination of codon 129 polymorphism in the prion protein gene (*PRNP*) was performed as described before [23].

### 2.5. Statistical Analysis

In order to determine associations between biomarkers and age at onset and sex, linear regression models (for continuous biomarkers) and logistic regression models (for categorical biomarkers) were used. Disease duration association with other variables was analysed with multivariate Cox proportional hazards models for survival analysis. Stratification based on the source of infection was not considered in any statistical analysis due to the low number of cases in two subgroups. Pearson correlation coefficients were used to assess associations between continuous biomarkers. All statistical analyses were conducted in R, except correlations, which were performed in GraphPad Prism 5 (GraphPad Software, San Diego, CA, USA).

### 2.6. Ethics

The study was conducted according to the revised Declaration of Helsinki and Good Clinical Practice guidelines, and was approved by local Ethics committees.

## 3. Results

### 3.1. Study Population

**Figure 1:**
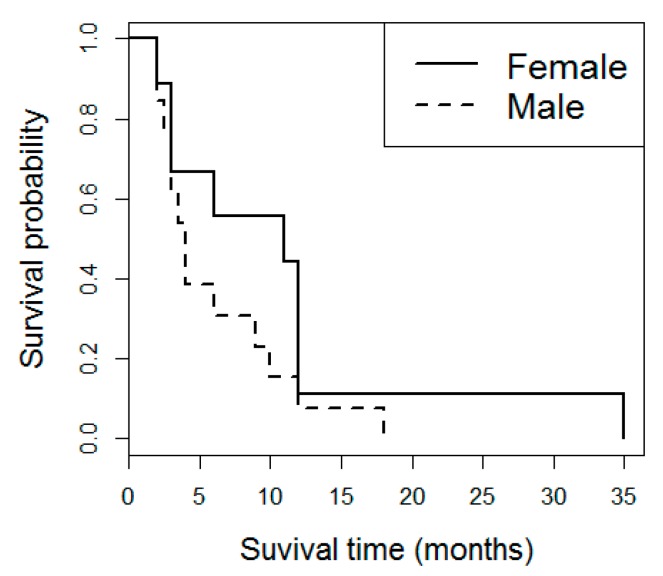
Disease duration in iCJD. Kaplan-Meier survival curves in iCJD stratified by sex.

Of the 23 iCJD cases included in the study, 14 were male and nine were female. Most iCJD cases (*n =* 20) were attributed to dura mater grafts, while two were associated with growth hormone therapy, and one was associated with corneal transplantation. Mean age at disease onset was 51.0 years old and mean disease duration was 7.9 months. Disease duration was significantly associated with age at onset and with sex (*p* < 0.01 in both). The hazard ratios were 1.0526 for the variable age and 4.2121 for the variable sex, with female patients showing increased disease duration (Figure 1). These association results with demographic parameters were not significantly altered when only dura matter grafts cases were considered.

Most of the cases (*n = *18) were methionine/methionine (MM) homozygous at codon 129 of the *PRNP* gene, while three were methionine/valine (MV) heterozygous and one was valine/valine (VV) homozygous (Table 1).

### 3.2. EEG and MRI

A positive EEG was recorded in 11 out of 22 cases with available data (sensitivity 50%). MRI results were recorded in 15 cases, being positive in 10 of them (sensitivity 66.6%). The degree of agreement between EEG and MRI outcome (number of positive or negative cases in both tests) was 60% (Table 2). EEG and MRI positivity was associated neither with age at onset nor with sex (*p* > 0.05).

### 3.3. CSF Biomarkers

14-3-3 was measured with WB (*n =* 23) and ELISA (*n =* 21). 14-3-3 ELISA quantification out-performed WB in terms of sensitivity (95.2% vs. 87%) with only one case below the established cut-off for sCJD (20000 AU/mL, [24]) and mean values of 93,047 AU/mL. t-tau (*n =* 23) displayed a sensitivity of 87% based on the established sCJD cut-off of 1300 pg/mL [13], with a mean concentration of 10,915 pg/mL. The addition to p-tau in the form of p-tau/t-tau ratio (*n =* 19) increased the sensitivity achieved by t-tau alone up to 94.7%, with only one case below the p-tau/t-tau cut-off (<0.075, [25]). Mean p-tau/t-tau ratio was 0.018. a-syn (*n =* 21) displayed a sensitivity of 90.5% based on the sCJD cut-off of 3300 pg/mL [26] with a mean concentration of 13,346 pg/mL. Nfl (*n =* 21) displayed a sensitivity of 85.7% using a cut-off of 7000 pg/mL [27] with a mean concentration of 12,986 pg/mL. The mean YKL-40 concentration (*n =* 21) was 441 ng/mL with a sensitivity of 76.2% based on a sCJD cut-off of 315 ng/mL [28]. Finally, RT-QuIC positive reactions were detected in 18 out of 21 cases (sensitivity of 85.7%) based on a cut-off of 10,000 relative fluorescent units (RFU) for sCJD [20]; mean RFU was 41,129 (Table 3). None of the CSF biomarkers were associated with age at onset or sex (*p* > 0.05 in all cases). Disease duration was not associated with any biomarker after controlling for the effects of age and sex.

### 3.4. Correlation between CSF Biomarkers

Correlations between continuous CSF biomarker data (biomarker concentrations for 14-3-3 (ELISA), t-tau, p-tau/t-tau ratio Nfl, a-syn, YKL-40, and RFU for RT-QuIC assay) were investigated. WB 14-3-3 data were not included due to the binary outcome of the assay.

Significant correlations were detected between 14-3-3 and t-tau (*ρ =* 0.57, *p =* 0.0067), 14-3-3 and p-tau/t-tau ratio (*ρ =* −0.52, *p =* 0.0208), t-tau and p-tau/t-tau ratio (*ρ =* −0.56, *p =* 0.0126), t-tau and a-syn (*ρ =* 0.70, *p <* 0.001), t-tau and Nfl (*ρ =* 0.48, *p =* 0.0259), t-tau and YKL-40 (*ρ =* 0.56, *p =* 0.0079), a-syn and Nfl (*ρ =* 0.45, *p =* 0.0396), a-syn and YKL-40 (*ρ =* 0.52, *p =* 0.0148), and Nfl and YKL-40 (*ρ =* 0.80, *p* < 0.001). RT-QuIC RFU did not correlate with any of the other biomarkers (*p* > 0.05 for all comparisons) (Table 4).

## 4. Discussion

The differential diagnosis of human prion diseases can be often challenging due to the phenotypic heterogeneity of the disease. Prion disease biomarker tests developed in the last two decades allow the detection of symptomatic sCJD and genetic prion disease cases, with *PRNP* sequence variations mimicking the sporadic phenotype (e.g., *PRNP*-E200K and *PRNP*-V210I) with high accuracy [27,29,30,31,32,33]. In contrast, very few studies have assessed the accuracy of the same biomarker tests in iCJD. Available studies described either very small numbers of cases, not necessarily representative of the overall cohort, or limited biomarker assays, mainly first-generation ones such as CSF 14-3-3 WB, EEG, or MRI [12,13,16].

Demographic and genetic characteristics of our iCJD cohort were similar to previous studies reviewing the iCJD outbreak [6]. Methionine homozygosity at codon 129 was over-represented in the present study (18 out of 22 cases), in agreement with the reported high prevalence of MM genotype in dura mater cases (80%) [6,11,16], which was indeed the source of infection most represented in our study population (87% of cases). Similar to other studies [12], mean age at onset in our iCJD cohort (51 years) was below that reported in sCJD (66 years old) [34]. Indeed, in the absence of recognized risk factors for iatrogenic transmission, iCJD may not be identified as a prion disease due to the significantly younger age at onset compared to sCJD [11]. We observed an association of disease duration with age at onset and also with sex, with female patients presenting longer survival times. This finding was previously described in sCJD and genetic prion diseases [35], as well as in iCJD [36]. Although a longer survival time in iCJD was also associated with MV heterozygosity at codon 129 and with cases caused by growth hormone treatment (compared to cases caused by dura mater implants) [36], we could not validate these findings probably due to the strong imbalance of our cohort regarding these variables.

In our biomarker panel study, we found the accuracy of EEG, MRI, and CSF 14-3-3 WB in iCJD to be broadly in line with what has been reported for sCJD. This finding is perhaps not surprising considering the similarity between the iCJD and sCJD clinical phenotypes [11]. When previously published data are simultaneously explored however, notable differences are observed between studied cohorts of iCJD. Regarding 14-3-3 detection by WB, a variety of sensitivities have been reported: 43% (*n =* 14, growth hormone-associated) [12], 75% (*n =* 20, source not indicated) [13], and 85% (*n =* 13, dura mater graft-associated) [16]. Other reports with one [37,38,39] or two iCJD cases [40] displayed 14-3-3 WB positivity in all samples. These differences may be related not to only the specific transmitted sCJD strain and the source of infection, but also to pre-analytical/analytical parameters as well as demographic/genetic characteristics of the studied cohorts. In our work, 14-3-3 WB sensitivity (87%) was at the upper range of previous iCJD studies, which is the same value as t-tau sensitivity. Importantly, 14-3-3 quantification with ELISA (sensitivity 95.2%) out-performed WB, displaying the highest sensitivity among the biomarkers herein tested. Since the development of the 14-3-3 ELISA, several studies in large cohorts of sCJD cases have demonstrated superior sensitivity compared to the WB method [24,40,41,42], but this is the first study to validate this observation in iCJD.

The value of EEG and MRI as diagnostic tools in iCJD was previously documented [11,14]. In a cohort of growth hormone-associated iCJD cases, EEG displayed a sensitivity of 71%, with the presence of MRI abnormalities in most of the cases [12]. CJD-associated changes on MRI were reported in two studied growth hormone-associated iCJD cases [43] and in 16 out of 22 (76%) dura matter-associated iCJD cases [16]. Case reports have also demonstrated the presence of typical abnormalities in growth hormone-related cases [44,45]. In our study, EEG and MRI displayed lower sensitivities than CSF biomarkers (50% and 66.6%, respectively) with poor agreement between both tests (60%). The low sensitivity of CJD-typical EEG findings is in line with other published data from CJD surveillance centres reporting sCJD cases [1]. In contrast, the herein reported sensitivity of MRI is lower than in sCJD cases [46,47,48]. On one side, this might be explained by the fact that most of the cases were reported to the reference centres before 2009, when standardized MRI criteria had not been established. On the other side, MRI data were available in only 15 out of the 23 iCJD cases; thus, caution should be taken when comparing our results with those from larger study cohorts.

Regarding next-generation CSF biomarkers, few data are available in the literature. In the present work, the addition of p-tau to t-tau quantification in the form of p-tau/t-tau ratio raised the sensitivity from 87% to 94.7%, which lies within the range of the sensitivities achieved in a large cohort of sCJD cases [29]. Interestingly, the cut-off utilised in the present study (<0.075) was generated from a cohort of sCJD and non-CJD cases wherein the control group included neurological controls and non-prion neurodegenerative diseases [25,29]. In the same cohort, exclusion of non-prion neurodegenerative diseases from the control group rendered a cut-off of <0.106 [29] that, when applied to the present iCJD study, boosts sensitivity to 100%.

Recently, CSF a-syn has been demonstrated to be a highly sensitive and specific biomarker for sCJD [26,30] and for gCJD associated with E200K and V210I mutations [30]. Herein, we also validated the value of this biomarker in the detection of iCJD cases with a sensitivity of 90.5%, which is only surpassed by the accuracies obtained for 14-3-3 ELISA and the p-tau/t-tau ratio. In contrast to surrogate markers of neuro-axonal degeneration such as tau and 14-3-3, a-syn is suggested to reflect synaptic loss, an early event in the pathology of neurodegeneration. Although synaptic and neuronal damage are common hallmarks in neurodegenerative diseases, CSF a-syn is highly specific for sCJD [30]. This is in contrast to CSF Nfl, another non-specific marker of axonal damage that is increased in several neurological conditions [49]. In our iCJD cohort, Nfl sensitivity (85.7%) was below that reported in sCJD cases [27,50,51]. YKL-40, a disease-specific marker of neuro- inflammation expressed in astrocytes, displayed the lowest sensitivity (76.2%) among CSF biomarkers herein tested. Elevated CSF YKL-40 levels are reported in sCJD, gCJD associated with E200K, and V210I mutations, and, to a lesser extent, in fatal familial insomnia [28]. CSF YKL-40 also appeared to be increased in other neurodegenerative dementias such as AD and FTD, but not in DLB [28,52,53].

The most recently updated diagnostic criteria for CJD incorporates the RT-QuIC assay as one of the CSF biomarker tests. This assay detects pathological prion protein in biofluids and has been reported to be highly sensitive and almost fully specific. We observed 85.7% sensitivity of RT-QuIC in our iCJD cohort, which is within the range of values described for sCJD [31,54]. Unfortunately, RT-QuIC results in iCJD are scarce in the literature. One study investigating iCJD reported positive RT-QuIC signal in two tested patients who received dura matter grafts [40]. Another report mentioned an RT-QuIC sensitivity of 67% in iCJD patients treated with cadaveric growth hormone although the data were not disclosed [55].

Investigation of potential associations between biomarkers in iCJD led us to the striking finding that RT-QuIC results do not correlate with any of the surrogate markers of prion pathology. While it is possible that RT-QuIC signal does not really reflect the degree of cerebral PrP pathogenesis, it may also be speculated that PrP seeding capacity for pathological conversion is not linked in a straightforward manner to neuronal damage and neuro-inflammation. By contrast, positive correlations were observed between several neuronal damage and neuro-inflammation markers, in line with those previously detected in sCJD [27,28,30,56,57]. Positive correlation between Nfl and t-tau with YKL-40 suggests a pathogenic association between astroglial activation and axonal injury in iCJD. Interestingly, Nfl vs.YKL-40 and t-tau vs. a-syn correlations were the most significant despite the fact that each pair of biomarkers is associated with distinct prion disease pathological features. While a positive correlation between Nfl and YKL-40 was previously detected in AD [58], this has not yet been investigated in prion diseases.

### Limitations of the Study

Due to the rarity of iCJD, a limited number of cases were available. Consequently, diagnostic accuracies may be partially biased due to case selection. Another important limitation is the impossibility to perform any subgroup statistical analyses based on infection source because the number of cases associated to growth hormone and corneal transplant are too low. Thus, data regarding source of infection remain purely descriptive.

## 5. Conclusions

In this study, we present the first simultaneous analysis of the available battery of prion disease biomarkers for iCJD. Among these, CSF biomarkers displayed greater sensitivity than EEG and MRI, with similar accuracies to those achieved in sCJD and in the most prevalent form of genetic prion disease (gCJD associated to E200K mutation) using the same cut-off points in the same ethnic populations (comparisons are summarized in Supplementary Table S2). Utilised CSF biomarkers covered the main pathological features of prion pathogenesis, including synaptic (a-syn) and neuronal damage (14-3-3, tau, Nfl), neuro-inflammation (YKL-40), and prion protein seeding and aggregation (RT-QuIC). Our study validates the use of the conventional panel of sCJD biomarkers for the diagnosis of iCJD. On one side, this would become useful in the event of the appearance of new iCJD cases (though not likely). In this regard, there is also the need to obtain data on prion biomarkers for acquired forms through blood transfusion from variant CJD donors in order to have more comprehensive information in the event of new iCJD. On the other side, the similarity between iCJD and sCJD in biomarker profiles and clinical phenotype also unveils the incapacity to distinguish sCJD from iCJD in potential situations of undiscovered sources of transmissibility, which compels the medical community to keep a strict and constant vigilance for future cases of iCJD to avoid mis-classification as sCJD.

## Figures and Tables

**Table 1:** Demographic, genetic, and clinical data in iatrogenic CJD. Source of infection, number of cases (*n*), sex (female (f)/male (m)), age at onset in years (mean with standard deviation (SD) and minimum and maximum years), codon 129 genotype (methionine (M), valine (V)), and disease duration in months (mean and standard deviation (SD), with minimum and maximum months) are indicated. NA: not available. * For one case, disease duration was not available.

			Age (years)	Codon 129 *PRNP*				Disease Duration (Months)
Source of Infection	n	Sex(f/m)	mean±SD (min-max)	MM	MV	VV	NA	Mean±SD (min-max)
Dura mater grafts	20	8/12	52 ± 15 (28-76)	16	3	0	1	8.0 ± 7.9 (2-35)
Growth hormone	2	0/2	39.5 ± 0.7 (39-40)	1	0	1	0	3*
Corneal transplant	1	1/0	45	1	0	0	0	11
Total	23	9/14	51 ± 15 (28-76)	18	3	1	1	7.9 ± 7.6 (2-35)

**Table 2:** Electroencephalogram and magnetic resonance imaging in iatrogenic CJD. Source of infection, electroencephalogram (EEG), and magnetic resonance imaging (MRI) outcome given as positive (Pos), negative (Neg), or not available (NA), and sensitivity of the biomarker test (%), are indicated.

	EEG				MRI			
Source of Infection	Pos	Neg	NA	Sensitivity (%)	Pos	Neg	NA	Sensitivity (%)
Dura mater grafts	9	10	1	47.4	9	4	7	69
Growth hormone	1	1	0	50	1	1	0	50
Corneal transplant	1	0	0	100	0	0	1	NA
Total	11	11	1	**50**	10	5	8	**66.6**

**Table 3:** Cerebrospinal fluid biomarkers in iatrogenic CJD. Cerebrospinal fluid (CSF) biomarker with cut-off point, source of infection, biomarker outcome given as positive, negative, or not available (NA), sensitivity of the biomarker test (%) with concentrations (mean with standard deviation (SD)), and minimum and maximum values are indicated. Data stratification based on infection source are only disclosed as descriptive information but no subgroup analysis was performed.

CSF Biomarker	Source of Infection	Positive	Negative	NA	Sensitivity (%)	Mean ± SD	Min-Max
14-3-3 western-blot	Dura mater grafts	17	3	0	85.0	-	-
	Growth hormone	2	0	0	100.0	-	-
	Corneal transplant	1	0	0	100.0	-	-
	Total	20	3	0	87.0	-	-
14-3-3 ELISA	Dura mater grafts	17	1	2	94.4	94,721±65,638	7677-254,152
(>20,000 AU/mL)	Growth hormone	2	0	0	100.0	56,120±36,753	36,753-75,488
	Corneal transplant	1	0	0	100.0	136,776	-
	Total	20	1	2	95.2	93,047±62,723	7677-254,152
t-tau	Dura mater grafts	17	3	0	85.0	10,374±9983	510-35,280
(>1300 pg/mL)	Growth hormone	2	0	0	100.0	19,356±22,299	3588-35124
	Corneal transplant	1	0	0	100.0	4833.0	-
	Total	20	3	0	87.0	10,915±10,822	510-35,280
p-tau/t-tau ratio	Dura mater grafts	16	1	3	94.1	0.018±0.025	0.002-0.094
(<0.075)	Growth hormone	1	0	1	100.0	0.012	-
	Corneal transplant	1	0	0	100.0	0.013	-
	Total	18	1	4	94.7	0.018±0.024	0.002-0.094
a-syn	Dura mater grafts	16	2	2	88.9	11,884±13,303	2075-45,698
(>3300 pg/ml)	Growth hormone	2	0	0	100.0	25,905±24,158	8823-42,987
	Corneal transplant	1	0	0	100.0	14,541	-
	Total	19	2	2	90.5	13,346±14,049	2075-45,698
Nfl	Dura mater grafts	15	3	2	83.3	12,900±7021	5311-29,856
(>7000 pg/mL)	Growth hormone	2	0	0	100.0	13,347±8892	7060-19,635
	Corneal transplant	1	0	0	100.0	14541	-
	Total	18	3	2	85.7	12,986±6776	5311-29,856
YKL-40	Dura mater grafts	13	5	2	72.2	417±205	127-887
(>315 ng/mL)	Growth hormone	2	0	0	100.0	630±303	416-845
	Corneal transplant	1	0	0	100.0	495.0	-
	Total	16	5	2	76.2	441±211	127-887
RT-QuIC	Dura mater grafts	16	2	2	88.9	44,180±16,679	10,000-65,000
(>10,000 RFU)	Growth hormone	1	1	0	50.0	21,910±16,843	10,000-33,820
	Corneal transplant	1	0	0	100.0	24,651	-
	Total	18	3	2	85.7	41,129±17,594	10,000-65,000

**Table 4:** Correlations between cerebrospinal fluid biomarkers. Cerebrospinal fluid (CSF) biomarkers correlations (biomarkers concentrations for 14-3-3 (ELISA), t-tau, p-tau/t-tau ratio Nfl, a-syn, YKL-40, and RFU for RT-QuIC assay). Pearson’s correlation coefficients are shown below the diagonal line (-) and *p* values are shown above. Statistically significant correlations (*p <* 0.05) are shown in bold.

	14-3-3	t-tau	p-tau/t-tau ratio	a-syn	Nfl	YKL-40	RT-QuIC
**14-3-3**	-	**0.0067**	**0.0208**	0.1822	0.8759	0.8064	0.7713
**t-tau**	**0.57**	-	**0.0126**	**<0.001**	**0.0259**	**0.0079**	0.4150
**p-tau/t-tau ratio**	**−0.52**	**−0.56**	-	0.0822	0.1938	0.3634	0.9507
**a-syn**	0.30	**0.70**	−0.41	-	**0.0396**	**0.0148**	0.1822
**Nfl**	0.04	**0.48**	−0.31	**0.45**	-	**<0.001**	0.9358
**YKL-40**	0.06	**0.56**	−0.22	**0.52**	**0.80**	-	0.1920
**RT-QuIC**	−0.07	−0.19	0.02	−0.29	−0.02	−0.30	-

## Supplementary Materials

The following are available online at https://www.mdpi.com/2218-273X/10/2/290/s1, Table S1: CJD-typical clinical symptoms reported at the time of lumbar puncture for each iCJD case involved in this study, Table S2: Sensitivities of the studied CSF biomarkers described in the present study for iCJD compared to the sensitivities of the same biomarkers reported in previous studies for sCJD and for gCJD associated with the E200K *PRNP* mutation in the same ethnic populations.
