# Supplementary material for: Diagnostic Accuracy of Prion Disease Biomarkers in Iatrogenic Creutzfeldt-Jakob Disease
Source: Biomolecules. 2020 Feb 12;10(2):290. doi: 10.3390/biom10020290 (PMC7072321; doi:10.3390/biom10020290)
Supplement: Supplementary file 1 [file biomolecules-10-00290-s001.pdf]

Supplementary Table 1. CJD-typical clinical symptoms reported at the time of lumbar puncture for each iCJD case involved in this study.

	Source of infection	First symptoms (onset)	Key clinical feature of CJD at time of lumbar puncture						
			cognitive impairment	cortical visual signs	cerebellar ataxia	extrapyramidal signs	pyramidal signs	myoclonus	akinetic mutism
**1**	**Dura mater grafts**	cerebellar ataxia	+	-	+	+	-	-	-
2	Dura mater grafts	cerebellar ataxia	+	-	+	-	+	+	-
3	Dura mater grafts	cerebellar ataxia	+	-	+	-	-	+	-
4	Dura mater grafts	cognitive impairment	+	-	+	-	-	+	-
5	Dura mater grafts	cognitive impairment	+	+	+	+	-	+	+
6	Dura mater grafts	cognitive impairment	+	+	+	-	-	+	-
7	Dura mater grafts	cerebellar ataxia	+	-	+	-	-	+	-
8	Dura mater grafts	cognitive impairment	+	-	-	+	+	+	-
9	Dura mater grafts	seizures	+	-	+	-	+	+	+
10	Dura mater grafts	cerebellar ataxia	+	-	+	-	-	+	-
11	Dura mater grafts	cerebellar ataxia	+	-	+	-	+	+	-
12	Dura mater grafts	personality changes, myoclonus	-	-	+	+	+	+	+
13	Dura mater grafts	cerebellar ataxia, cognitive impairment	+	-	+	-	-	-	-
14	Dura mater grafts	cognitive impairment	+	-	-	-	-	-	-
15	Dura mater grafts	cognitive impairment, visual symptoms, headaches	+	+	-	+	-	+	-
16	Dura mater grafts	visual symptoms, dysarthria, headaches	+	+	+	+	-	+	-
17	Dura mater grafts	seizures	The patient presented seizures at clinical onset which were recurrent and did not respond to treatment. Other distinct CJD-typical symptoms might habe been superimposed						
18	Dura mater grafts	cerebellar ataxia	+	+	+	-	-	+	-
19	Dura mater grafts	extrapyramidal disease and progressive dementia	+	-	-	+	+	+	-
20	Dura mater grafts	cognitive impairment	+	-	-	+	-	+	+
21	Corneal transplant	cerebellar ataxia	+	-	+	+	+	+	-
22	Growth hormone	cerebellar ataxia	-	-	+	-	+	+	-
23	Growth hormone	ataxia and lower limb dysaesthesia possibly related to iCJD onset	Further information was not available						

Supplementary Table 2. Sensitivities of the studied CSF biomarkers described in the present study for iCJD compared to the sensitivities of the same biomarkers reported in previous studies for sCJD and for gCJD associated with the E200K *PRNP* mutation in the same ethnic populations.

	sCJD		gCJD-E200K		iCJD
	**Sensitivity (%)**	References	Sensitivity (%)	References	Sensitivity (%)
Western-blot 14-3-3	77 - 85	[1–3]	64 - 89	[1,3,4]	87
ELISA 14-3-3	88 - 97	[5–7]	NA	NA	95
t-tau	86 - 97	[1,2,6]	75 - 85	[1,4]	87
p-tau/t-tau ratio	97 - 100	[6,8,9]	99	[8]	95
a-syn*	86	[10]	NA	NA	91
Nfl	86 - 100	[11]	95	[11]	86
YKL-40	85	[12]	82	[12]	76
RT-QuIC	80 - 85	[1,3,13]	100	[1,3,13]	86

*Comparison only with studies using the same methodological platform.

References

1. Lattanzio, F.; Abu-Rumeileh, S.; Franceschini, A.; Kai, H.; Amore, G.; Poggiolini, I.; Rossi, M.; Baiardi, S.; McGuire, L.; Ladogana, A.; et al. Prion-specific and surrogate CSF biomarkers in Creutzfeldt-Jakob disease: diagnostic accuracy in relation to molecular subtypes and analysis of neuropathological correlates of p-tau and Aβ42 levels. *Acta Neuropathol.* **2017**, *133*, 559–578.
2. Sanchez-Juan, P.; Green, A.; Ladogana, A.; Cuadrado-Corrales, N.; Sáanchez-Valle, R.; Mitrováa, E.; Stoeck, K.; Sklaviadis, T.; Kulczycki, J.; Hess, K.; et al. CSF tests in the differential diagnosis of Creutzfeldt-Jakob disease. *Neurology* **2006**, *67*, 637–643.
3. Abu-Rumeileh, S.; Capellari, S.; Stanzani-Maserati, M.; Polischi, B.; Martinelli, P.; Caroppo, P.; Ladogana, A.; Parchi, P. The CSF neurofilament light signature in rapidly progressive neurodegenerative dementias. *Alzheimer's Res. Ther.* **2018**, *10*.
4. Ladogana, A.; Sanchez-Juan, P.; Mitrova, E.; Green, A.; Cuadrado-Corrales, N.; Sanchez-Valle, R.; Koscova, S.; Aguzzi, A.; Sklaviadis, T.; Kulczycki, J.; et al. Cerebrospinal fluid biomarkers in human genetic transmissible spongiform encephalopathies. *J Neurol* **2009**, *256*, 1620–1628.
5. Schmitz, M.; Ebert, E.; Stoeck, K.; Karch, A.; Collins, S.; Calero, M.; Sklaviadis, T.; Laplanche, J.L.; Golanska, E.; Baldeiras, I.; et al. Validation of 14-3-3 Protein as a Marker in Sporadic Creutzfeldt-Jakob Disease Diagnostic. *Mol. Neurobiol.* **2016**, *53*, 2189–2199.
6. Leitão, M.J.; Baldeiras, I.; Almeida, M.R.; Ribeiro, M.H.; Santos, A.C.; Ribeiro, M.; Tomás, J.; Rocha, S.; Santana, I.; Oliveira, C.R. Sporadic Creutzfeldt-Jakob disease diagnostic accuracy is improved by a new CSF ELISA 14-3-3?? assay. *Neuroscience* **2016**, *322*, 398–407.
7. Gmitterová, K.; Heinemann, U.; Bodemer, M.; Krasnianski, A.; Meissner, B.; Kretzschmar, H.A.; Zerr, I. 14-3-3 CSF levels in sporadic Creutzfeldt-Jakob disease differ across molecular subtypes. *Neurobiol. Aging* **2009**, *30*, 1842–1850.
8. Llorens, F.; Schmitz, M.; Karch, A.; Cramm, M.; Lange, P.; Gherib, K.; Varges, D.; Schmidt, C.; Zerr, I.; Stoeck, K. Comparative analysis of cerebrospinal fluid biomarkers in the differential diagnosis of neurodegenerative dementia. *Alzheimers. Dement.* **2015**, 1–13.
9. Riemenschneider, M.; Wagenpfeil, S.; Vanderstichele, H.; Otto, M.; Wiltfang, J.; Kretzschmar, H.; Vanmechelen, E.; Förstl, H.; Kurz, A. Phospho-tau/total tau ratio in cerebrospinal fluid discriminates Creutzfeldt–Jakob disease from other dementias. *Mol. Psychiatry* **2003**, *8*, 343–347.

10. Schmitz, M.; Villar-Piqué, A.; Llorens, F.; Gmitterová, K.; Hermann, P.; Varges, D.; Zafar, S.; Lingor, P.; Vanderstichele, H.; Demeyer, L.; et al. Cerebrospinal Fluid Total and Phosphorylated α-Synuclein in Patients with Creutzfeldt–Jakob Disease and Synucleinopathy. *Mol. Neurobiol.* **2019**, *56*, 3476–3483.
11. Zerr, I.; Schmitz, M.; Karch, A.; Villar-Piqué, A.; Kanata, E.; Golanska, E.; Díaz-Lucena, D.; Karsanidou, A.; Hermann, P.; Knipper, T.; et al. Cerebrospinal fluid neurofilament light levels in neurodegenerative dementia: Evaluation of diagnostic accuracy in the differential diagnosis of prion diseases. *Alzheimer's Dement.* **2018**, *14*, 751–763.
12. Llorens, F.; Thüne, K.; Tahir, W.; Kanata, E.; Diaz-Lucena, D.; Xanthopoulos, K.; Kovatsi, E.; Pleschka, C.; Garcia-Esparcia, P.; Schmitz, M.; et al. YKL-40 in the brain and cerebrospinal fluid of neurodegenerative dementias. *Mol. Neurodegener.* **2017**, *12*.
13. Cramm, M.; Schmitz, M.; Karch, A.; Mitrova, E.; Kuhn, F.; Schroeder, B.; Raeber, A.; Varges, D.; Kim, Y.S.; Satoh, K.; et al. Stability and Reproducibility Underscore Utility of RT-QuIC for Diagnosis of Creutzfeldt-Jakob Disease. *Mol. Neurobiol.* **2016**, *53*, 1896–1904.
